# Perineal Body Anatomy in Primiparas Assessed by Three-Dimensional Endovaginal and Endoanal Ultrasound: A Reliability Study

**DOI:** 10.1007/s00192-025-06379-5

**Published:** 2025-10-24

**Authors:** Hanne Sether Lilleberg, Franziska Siafarikas, Marianne Starck, Emilia Rotstein, Kari Bø, Marie Ellström Engh

**Affiliations:** 1 Department of Obstetrics and Gynecology, Akershus University Hospital, Lørenskog, Norway; 2 Faculty of Medicine, University of Oslo, Lørenskog, Norway; 3 Pelvic Floor Center, Department of Surgery, Skåne University Hospital, Malmö, Sweden; 4 Department of Clinical Science, Intervention and Technology (CLINTEC), Institution of obstetrics and gynecology, Karolinska Institute, Huddinge, Sweden; 5 Karolinska Pelvic Floor Centre, Department of Gynecology and Reproductive Medicine, Karolinska University Hospital, Stockholm, Sweden; 6 Department of Sports Medicine, Norwegian School of Sports Sciences, Oslo, Norway

**Keywords:** 3D endoanal ultrasound, 3D endovaginal ultrasound, Pelvic floor muscles, Perineal body, Reliability

## Abstract

**Introduction and Hypothesis:**

The integrity of the perineal body (PB) is essential for pelvic floor function. Anatomical deviations in the PB anatomy after childbirth can lead to pelvic floor symptoms, but the reliability of ultrasound assessments for these deviations is unclear. This study evaluates the intra- and inter-rater reliability of detecting deviations in the muscles fusing into the PB and measuring its size using three-dimensional endovaginal (3D EVUS) and endoanal ultrasound (3D EAUS) 1 year postpartum.

**Methods:**

Forty primiparous women underwent 3D EVUS and 3D EAUS 1 year after birth. The transverse perineal, puboperinealis, and puboanalis muscles were identified where they fuse into the PB. Deviations were defined as discontinuities in or absence of muscle fibers on the right, left, or center. Measurements of PB height, length, and area were taken in the mid-sagittal plane. Intra- and inter-rater reliability for muscle deviations were assessed using percentage agreement and Cohen’s kappa, while PB size measurements were evaluated using the intra-class correlation coefficient.

**Results:**

Kappa values indicated excellent intra- and inter-rater reliability for the transverse perineal muscle (*κ* = 0.881, *κ* = 0.871) and puboperinealis muscle (*κ* = 0.849, *κ* = 0.810) in 3D EVUS. For the puboanalis muscle, intra-rater reliability was excellent (*κ* = 1.00) and inter-rater reliability was good (*κ* = 0.658). Similar kappa values were found using 3D EAUS. Percentage agreement for deviations ranged from 90% to 100% for both ultrasound methods. PB measurement also showed good to excellent reliability: 0.76-0.97 for 3D EVUS and 0.90–0.99 for 3D EAUS.

**Conclusion:**

3D EVUS and 3D EAUS are reliable methods for assessing PB muscle deviations and its size 1 year postpartum.

## Introduction

Ultrasound has improved our understanding of childbirth-related pelvic floor injuries and their association with pelvic floor dysfunction, with obstetric anal sphincter injuries (OASI) and levator ani avulsion serving as examples [1, 2]. A similar application may be possible for injuries to the perineal body complex. The perineal body is at the lowest level of the pelvic floor, at the pelvic outlet, where the perineal structures experience maximum strain during vaginal childbirth. Consequently, it is the most common site of childbirth-related pelvic floor injuries [3]. The perineal body is a fibromuscular structure where the transverse perineal, puboperinealis, puboanalis, and external anal sphincter fuse [4–8]. It is often described as the “anchor” of the pelvic floor, highlighting its central role in pelvic floor function [5, 9]. We have recently demonstrated that primiparous women with deviations in the muscles fusing into the perineal body, as detected by three-dimensional endovaginal (3D EVUS) and endoanal ultrasound (3D EAUS) 1 year after birth, reported more pelvic floor symptoms, including pain, sexual dysfunction, vaginal flatulence, and difficulties with bowel emptying compared to women without such deviations [10].

Ensuring the reliability of the 3D EVUS and 3D EAUS assessment of the perineal body anatomy is the key requirement for both research and clinical application. 3D EVUS has been shown to identify the muscles fusing into the perineal body in nulliparous women [11]. However, there are no studies to our knowledge that have assessed the reliability of detecting deviations in these muscles postpartum.

This study aims to assess the intra- and inter-rater reliability of detecting deviations in the muscles fusing into the perineal body and measuring perineal body size using 3D EVUS and 3D EAUS 1 year after childbirth.

## Material and Methods

This study was conducted during 01/2023 and 04/2023. Women included in this study participated in the “The Perineum study,” a prospective cohort study that aimed to investigate the consequences of second-degree perineal tears 1 year after delivery [3]. To be included in the “Perineum Study,” participants had to have a singleton pregnancy and understand the Norwegian language. Immediate exclusion criteria were genital mutilation and, for multiparous women, prior cesarean section or a third- or fourth-degree perineal tear. Later exclusion criteria included delivery at another institution, perineal tears not subclassified, and intrauterine fetal death. For the present study, only primiparous women were eligible.

Our research team developed an analysis protocol to assess perineal anatomy following vaginal childbirth. Identification of the muscles fusing into the perineal body was guided by earlier publications from Shobeiri and Santoro on nulliparous women [11, 12]. Subsequently, a protocol for detecting muscle deviations was created by comparing ultrasound volumes of primiparous women who gave birth by cesarean section to those who had delivered vaginally. The protocol also included measurements of perineal body size.

### Outcome Measure

The outcome measure of this study is the intra- and inter-rater reliability of muscle deviations in the muscles fusing into the perineal body and measurements of the perineal body size using 3D EVUS and 3D EAUS 1 year after delivery.

### Study Sample

The study sample included 40 participants from “The Perineum study,” 20 primiparous women who gave birth through a cesarean delivery, and 20 primiparous women with vaginal deliveries.

### Volume Acquisitions

The ultrasound examinations were performed 1 year postpartum by a single investigator (HSL) using a 3D BK-5000 machine (BK Medical, Herlev, Denmark) with a 9038 3D ART transducer. This is a high-resolution probe featuring a linear array of ultrasound crystals with a built-in system that rotates 360° to generate 3D imaging. A frequency of 12 MHz was applied during the scans. The participants were asked to void as needed. The ultrasound examination was performed in a dorsal lithotomy position. The probe, covered with gel and a single-use cover, was inserted into the vagina. To ensure correct probe placement, the pubic symphysis and bladder served as anterior landmarks, while the puborectalis muscle and the perineal body were used as posterior landmarks [11]. The acquisition of ultrasound volumes was executed through a 60-s pre-programmed automated sequence. Subsequently, the probe was inserted into the anal canal, with the perineal body and the puborectalis muscle serving as anterior and posterior landmarks, respectively. Another automated 60-s scan was then performed. All ultrasound data were digitally stored for offline evaluation.

### Offline Analysis

For processing the volumes, the BK3D-Viewer v.9.0.20 software medical system was utilized. The analysis was independently conducted by the first author (HSL) and the third author (MS). HSL is a resident in obstetrics and gynecology with a special focus on ultrasound diagnostics, while MS is a senior colorectal surgeon responsible for the development of a scoring system for anal sphincter injuries using 3D EAUS [13]. She has extensive experience in 3D EVUS interpretation [14].

**Fig. 1:**
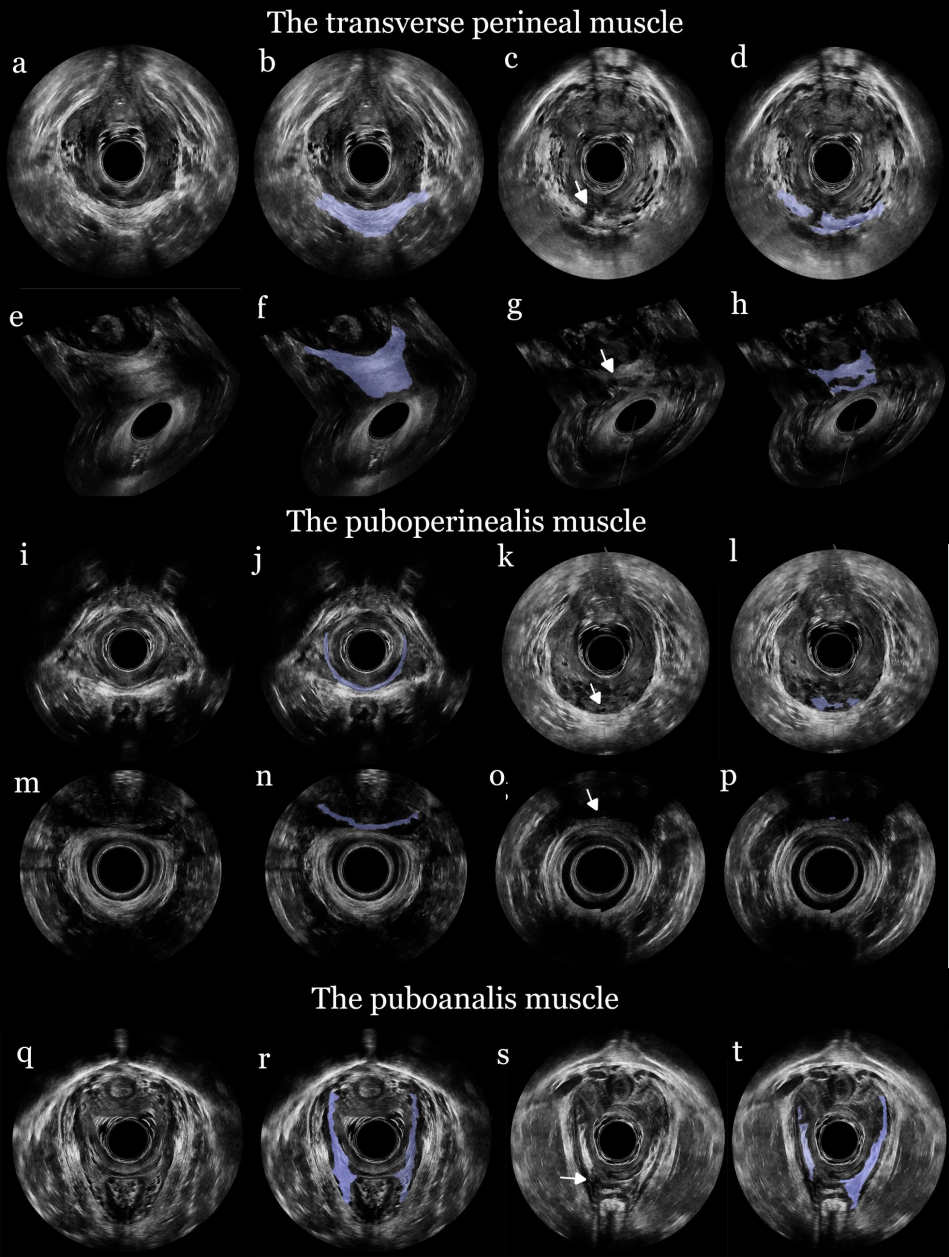
Three-dimensional ultrasound of the transverse perineal, puboperinealis, and puboanalis muscles. The transverse perineal muscle: In the three-dimensional endovaginal ultrasound in the axial plane: **a** Normal muscle; **b** Normal muscle, highlighted in blue; **c** An arrow pointing to a central deviation in the muscle; **d** Fragments of the remaining muscle, highlighted in blue. In the three-dimensional endoanal ultrasound in the coronal plane: **e** Normal muscle; **f** Normal muscle, highlighted in blue; **g** An arrow pointing to a central deviation in the muscle; **h** Fragments of the remaining muscle, highlighted in blue. The puboperinealis muscle: In the three-dimensional endovaginal ultrasound in the axial plane: **i** Normal muscle; **j** Normal muscle, highlighted in blue; **k** An arrow pointing to a central deviation in the muscle; **l** Fragments of the remaining muscle, highlighted in blue. In the three-dimensional endoanal ultrasound in the axial plane: **m** Normal muscle; **n** Normal muscle, highlighted in blue; **o** An arrow pointing to an absent muscle; **p** Fragments of the remaining muscle, highlighted in blue. The puboanalis muscle: In the three-dimensional endovaginal ultrasound in the axial plane: **q** Normal muscle; **r** Normal muscle, highlighted in blue; **s** An arrow pointing to a right deviation in the muscle; **t** Fragments of the remaining muscle, highlighted in blue

In the 3D EVUS volumes, the axial plane was first adjusted to align the pubic symphysis and anal canal as anterior–posterior reference points, ensuring optimal visualization of the volume. The transverse perineal, puboperinealis, and puboanalis muscles were identified where they fuse into the perineal body at a distal and a proximal level. The *distal level* included the transverse perineal and the puboperinealis muscles. The transverse perineal muscle was seen as a hyperechoic band oriented between the ischial tuberosities (Fig. 1). The puboperinealis muscle appeared as a U-shaped hyperechoic structure adjacent to the vagina and positioned anteriorly to the transverse perineal muscle (Fig. 1). Advancing proximally in the axial plane, the puboperinealis and the puboanalis muscles were identified at a *proximal level*. At this level, the puboperinealis muscle maintained its U-shaped hyperechoic band between the vagina and anus. Laterally, the puboanalis muscle was observed as a hyperechoic triangular structure inserting fibers at the distal part of the external sphincter muscle (Fig. 1).

In the 3D EAUS volumes, the axial plane was adjusted to ensure that the internal and external sphincter appeared as evenly circular structures. The transverse perineal muscle appeared as a hyperechoic band adjacent and anterior to the external sphincter muscle (Fig. 1). Positioned anteriorly, the puboperinealis exhibited its characteristic U-shape (Fig. 1). The puboanalis muscle was not possible to visualize in the 3D EAUS volumes and therefore excluded from this analysis.

Muscle deviations in the transverse perineal and the puboperinealis muscles were defined as a discontinuity or absence of the muscle structure on either the right or left side or centrally in the images (Fig. 1) [10]. For the puboanalis muscle, deviations were determined on the basis of its continuity or absence in the left or right portions of the images (Fig. 1) [10]. Once the respective muscle was identified at the correct level in the axial plane, the full 3D volume, including all three orthogonal planes (axial, sagittal, and coronal), was analyzed to determine the presence of a deviation. A muscle deviation was confirmed only if a discontinuity was evident in at least two of these planes. If artifacts, such as air bubbles, interfered with the visualization of a muscle, the volume was designated as poor quality for the respective muscle but was still included in the reliability assessment.

**Fig. 2:**
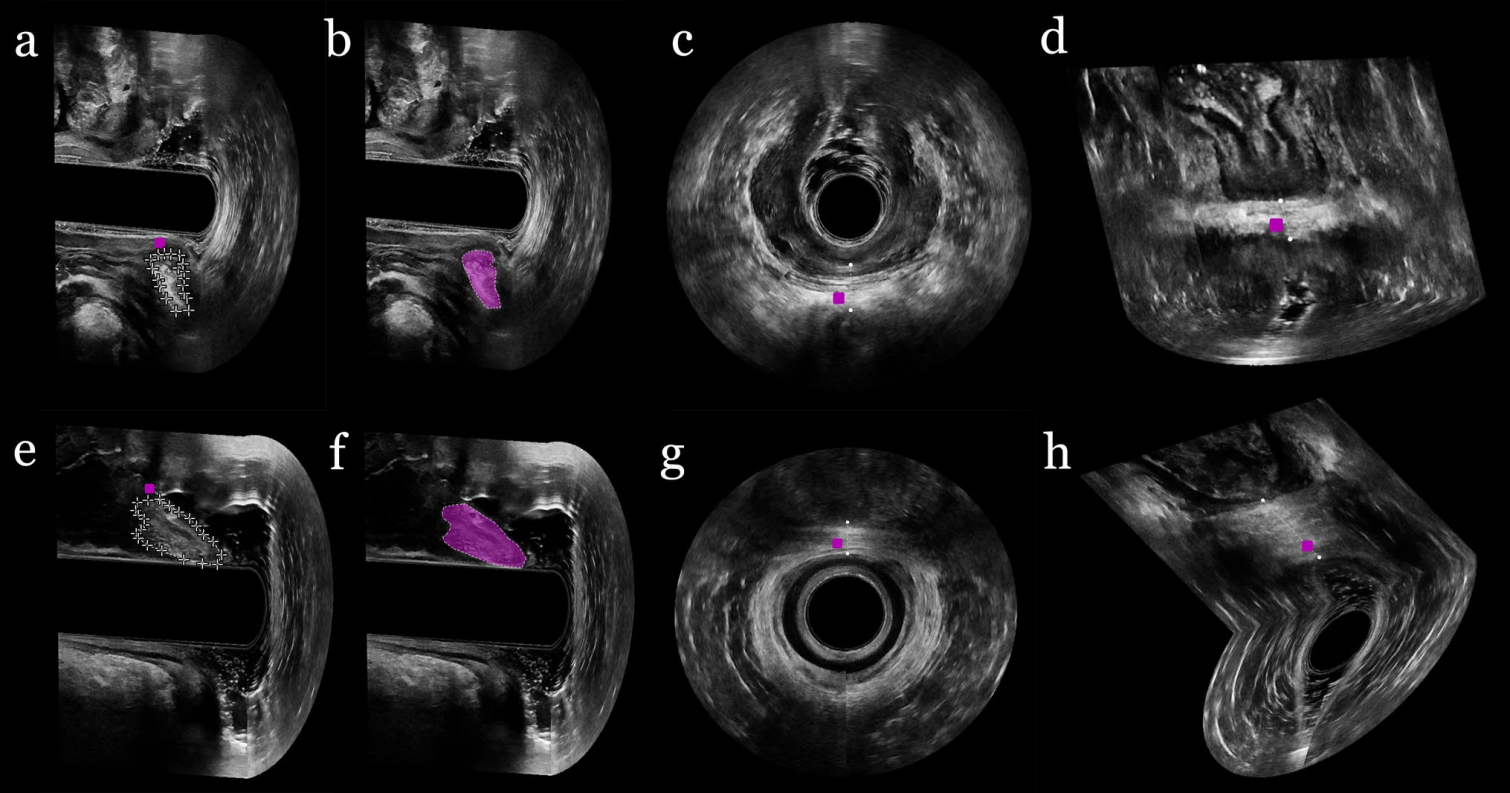
Three-dimensional ultrasound imaging of the perineal body. Three-dimensional endovaginal ultrasound: **a** Area in the sagittal plane; **b** Area in the sagittal plane, highlighted in pink; **c** The dotted line showing the height in the axial plane; **d** The dotted line showing the length in the coronal plane. Three-dimensional endoanal ultrasound: **e** Area in the sagittal plane; **f** Area in the sagittal plane, highlighted in pink; **g** The dotted line showing the height in the axial plane; **h** The dotted line showing the length in the coronal plane

The perineal body was defined as a hyperechoic heart-shaped structure located between the anal canal and the vagina, including fibers from the external sphincter, transverse perineal, puboperinealis, and puboanalis muscles. Length, height, and area were obtained in the midsagittal plane in 3D EVUS and 3D EAUS (Fig. 2). Length was measured as the longest proximodistal diameter and height was measured as the longest anteroposterior diameter perpendicular to the length. The area was measured as the size around the hyperechoic structure. The axial and coronal planes were used to help identify the borders.

For intra-rater reliability, the first investigator (HSL) analyzed the ultrasound volumes in a random order at two separate times (1a and 1b), with at least a 2-week interval between analyses. For the inter-rater reliability, the second investigator (MS) performed an independent analysis (2), and these results were compared to the initial analysis by the first investigator (1a). Both investigators were blinded to obstetric data and previous analysis. A maximum of five examinations were analyzed per session.

### Statistical Analysis

Statistical analysis was performed using SPSS version 29. For deviations in the muscles, percentage agreement was calculated as the proportion of the same observations divided by the total number of observations multiplied by 100. Intra- and inter-rater reliability were analyzed with Cohen’s kappa index and reported with 95% confidence intervals (CI). Kappa values of less than 0.20 were considered poor, 0.21–0.40 minimal, 0.41–0.60 moderate, 0.61–0.80 good, and 0.81–1.0 excellent reliability [15]. Descriptive statistics for continuous data were given as mean values with a standard deviation. Intra- and inter-rater reliability for perineal body measurements were determined using the intraclass correlation coefficient (ICC) with 95% CI (ICC 0.0–0.50 poor, 0.51–0.75 moderate, 0.76–0.90 good, 0.91–1.00 excellent reliability) [16]. Limits of agreement and bias were calculated [17].

### Ethics

The study has received ethical approval. Written informed consent was obtained from all participants before entering the study.

## Results

Table 1 presents background and delivery data. Of the vaginally delivered participants, 15% had a vacuum delivery and 45% had a second-degree perineal tear. Episiotomy was performed in 25% of vaginal deliveries.

**Table 1:** Background information and obstetric data

Variable	Cesarean section *n* = 20	Vaginal delivery *n* = 20
Age in years, mean (SD)	**33.6 (3.4)**	**31.8 (2.7)**
BMI in kg/m^2^, mean (SD)	**25.7 (5.7)**	**24.4 (4.4)**
Stages of labor in the CS group, *n* (%)		
Elective	**5 (25)**	
First stage	**12 (60)**	
Second stage	**3 (15)**	
Delivery method, *n* (%)		
Non-instrumental vaginal delivery		**17 (85)**
Vacuum delivery		**3 (15)**
Perineal tear^†^, *n* (%)		
Grade 1		**6 (30)**
Grade 2		**9 (45)**
Grade 3 or 4		**0 (0)**
Lateral episiotomy^‡^		**5 (25)**

^*^ *BMI* body mass index, *CS* cesarean section, *n* number

^**†**^ Perineal tears classified according to RCOG classification [18]

^‡^ Women with episiotomies may have had an additional perineal tear

### Muscle Deviations

The number of detected deviations in the transverse perineal, puboperinealis, and puboanalis muscles identified by 3D EVUS and 3D EAUS categorized by mode of delivery are presented in Table 2. In the cesarean section group, all muscles appeared intact in both 3D EVUS and 3D EAUS volumes, except in one woman where the puboperinealis muscle was only identified in the 3D EVUS. Among participants who had delivered vaginally, the most common deviations were noted in the transverse perineal and puboperinealis muscles at the distal level.

**Table 2:** Number of detected deviations in the transverse perineal, puboperinealis, and puboanalis muscles using three-dimensional endovaginal (3D EVUS) and endoanal ultrasound (3D EAUS), stratified by mode of delivery

	Cesarean section *n* = 20			Vaginal delivery *n* = 20		
Investigator^†^	1a	1b	2	1a	1b	2
*3D EVUS*						
**Distal level**						
Transverse perineal						
Intact	20	20	20	10	10	12
Discontinuity right	0	0	0	3	3	2
Discontinuity left	0	0	0	2	2	2
Discontinuity central	0	0	0	3	4	3
Poor quality	0	0	0	2	1	1
Puboperinealis						
Intact	20	20	20	6	7	5
Not visible	0	0	0	10	10	9
Poor quality	0	0	0	4	3	6
**Proximal level**						
Puboperinealis						
Intact	20	20	20	18	16	18
Discontinuity right	0	0	0	1	1	1
Discontinuity left	0	0	0	1	1	1
Not visible	0	0	0	0	2	0
Puboanalis						
Normal	20	20	20	18	18	19
Discontinuity right	0	0	0	1	1	1
Discontinuity left	0	0	0	1	1	0
*3D EAUS*						
Transverse perineal						
Intact	20	20	20	15	14	15
Discontinuity right	0	0	0	0	0	1
Discontinuity left	0	0	0	1	1	1
Discontinuity central	0	0	0	3	4	2
Not visible	0	0	0	1	1	1
Puboperinealis						
Intact	19	19	19	8	9	11
Not visible	1	1	1	12	11	9

^*^ *n* number

^†^ Investigator 1a and 1b refer to the first and second analyses conducted by the first investigator, while investigator 2 represents the single examination performed by the second investigator

Table 3 presents the percentage agreement and Cohen’s kappa values for intra- and inter-rater reliability in detecting muscle deviations. Intra-rater reliability showed percentage agreement ranging from 92 to 100% in 3D EVUS volumes and 95% to 97% in 3D EAUS volumes. Most kappa values indicated excellent reliability, except for the puboperinealis muscle at the proximal level in the 3D EVUS volumes, which had a Kappa value of 0.652, reflecting good reliability. For inter-rater reliability, the percentage agreement ranged from 90% to 100% in 3D EVUS and 92% to 95% in 3D EAUS volumes. Kappa values indicated excellent reliability for all muscles in both imaging modalities, except for the puboanalis muscle in 3D EVUS volumes, which had a kappa value of 0.658, indicating good reliability. Subgroup analysis according to delivery mode did not substantially affect the intra- or inter-rater Kappa values in the vaginal delivery group.

**Table 3:** Intra- and inter-rater reliability for detection deviations in the transverse perineal, puboperinealis, and puboanalis muscles using three-dimensional endovaginal (3D EVUS) and endoanal ultrasound (3D EAUS)

	Intra-rater (1a and 1b)†		Inter-rater (1a and 2)†	
	Percentage agreement	Cohen’s kappa (95% CI)	Percentage agreement	Cohen’s kappa (95% CI)
**3D EVUS**				
**Distal level**				
Transversus perineal	97%	0.881 (0.799–0.963)	95%	0.871 (0.782–0.960)
Puboperinealis	92%	0.849 (0.769–0.929)	90%	0.810 (0.726–0.894)
**Proximal level**				
Puboperinealis	95%	0.652 (0.427–0.877)	100%	1 (1.0–1.0)
Puboanalis	100%	1 (1.0–1.0)	97%	0.658 (0.342–0.974)
**3D EAUS**				
Transversus perineal	97%	0.899 (0.799–0.999)	95%	0.782 (0.634–0.930)
Puboperinealis	95%	0.886 (0.808–0.964)	92%	0.818 (0.719–0.917)

^*^ *CI* confidence interval

^†^ 1a and 1b refer to the first and second analyses conducted by the first investigator, while investigator 2 represents the single examination performed by the second investigator

### Perineal Body

Table 4 presents the mean length, height, and area of the perineal body by both investigators using 3D EVUS and 3D EAUS, stratified by mode of delivery. Across all analyses, perineal body measurements were consistently larger in 3D EAUS compared to 3D EVUS. Intra-rater reliability was excellent in both imaging modalities with ICC values ranging from 0.93–0.99 (Table 4). Inter-rater reliability was good for length, height, and area in 3D EVUS, with ICC values of 0.76, 0.85, and 0.87, respectively, and excellent for all measurements in 3D EAUS. A significant measurement bias was observed for perineal body height in 3D EVUS, where investigator 1 reported overall higher values than investigator 2.

**Table 4:** Mean perineal body size, stratified by investigator and mode of delivery, along with intra- and inter-rater reliability for perineal body measurements assessed using three-dimensional endovaginal (3D EVUS) and endoanal ultrasound (3D EAUS)

	Cesarean section			Vaginal delivery			Intra-rater, 1a and 1b^†^					Inter-rater, 1a and 2^†^				
	1a^†^	1b^†^	2^†^	1a^†^	1b^†^	2^†^	Limit of agreement					Limit of agreement				
							Mean difference (95% CI)	Bias (95% CI)	Lower	Upper	ICC	Mean difference (95% CI)	Bias (95% CI)	Lower	Upper	ICC
**3D EVUS**																
Length, mm	11.25 (2.12)	10.90 (2.07)	11.85 (1.92)	12.20 (2.09)	12.05 (2.25)	12.05 (1.90)	0.25 (−0.09; 0.59)	−0.41 (−0.58; −0.25)	−1.96	2.46	0.93	−0.23 (−0.31; 0.77)	0.15 (−0.16; 0.46)	−3.71	3.26	0.76
Height, mm	6.70 (1.55)	6.65 (1.84)	6.60 (1.18)	8.05 (2.30)	7.70 (2.40)	6.85 (1.78)	0.20 (−0.03; 0.43)	−0.06 (−0.17; 0.05)	−1.29	1.69	0.97	0.65 (0.28; 1.02)	0.34 (0.14; 0.54) ‡	−1.72	3.02	0.85
Area, cm^2^	0.57 (0.17)	0.53 (0.19)	0.54 (0.14)	0.66 (0.20)	0.66 (0.22)	0.56 (0.18)	0.02 (−0.22; 0.26)	−0.12 (−0.23; 0.00)	−0.13	0.17	0.93	0.06 (−0.28; 0.40)	0.19 (0.00; 0.38)	−0.16	0.28	0.87
**3D EAUS**																
Length, mm	17.15 (3.15)	16.95 (3.26)	16.03 (3.10)	16.30 (3.22)	16.00 (3.43)	15.30 (2.90)	0.25 (−0.07; 0.58)	−0.05 (−0.15; 0.05)	−1.82	2.32	0.97	0.70 (0.23; 1.16)	0.04 (−0.11; 0.20)	−2.22	3.62	0.93
Height, mm	9.0 (1.21)	9.0 (1.13)	8.50 (1.23)	7.25 (1.88)	7.25 (1.97)	7.60 (1.66)	0.0 (−0.12; 0.12)	−0.05 (−0.11; 0.02)	−0.77	0.77	0.99	−0.38 (−0.69; −0.07)	0.06 (−0.13; 0.24)	−2.34	1.59	0.90
Area, cm^2^	1.07 (0.21)	1.06 (0.22)	0.97 (0.20)	0.85 (0.25)	0.83 (0.25)	0.85 (0.25)	0.02 (0.00; 0.02)	−0.05 (−0.10; 0.00)	-0.06	0.1	0.99	−0.01 (−0.03; 0.01)	−0.03 (−0.11; 0.05)	−0.14	0.13	0.98

^*^ *CI* confidence interval, *ICC* intraclass coefficient

^†^ Investigator 1a and 1b refer to the first and second analyses conducted by the first investigator, while investigator 2 represents the single examination performed by the second investigator

^‡^ Significant, *p* value <0.05

## Discussion

The main finding of this study is that 3D EVUS and 3D EAUS are reliable methods to identify deviations in the transverse perineal, puboperinealis, and puboanalis muscles in the area where they fuse into the perineal body, and to obtain measurements of the perineal body size 1 year after birth.

### Muscle Deviations

The identification of normal muscles in our protocol was guided by imaging studies on nulliparous women showing that the transverse perineal, puboperinealis, and puboanalis muscles can be reliably identified [11, 12]. We defined a deviation in the muscle as either an absence or discontinuity at the expected anatomical site based on definitions of abnormal ultrasound findings of the levator ani muscle at its insertion to the pubic bone and defects in the anal sphincter complex using 3D EVUS and 3D EAUS [13, 19]. When developing our analysis protocol prior to this study, women who had delivered by cesarean section were used as reference for normal anatomy. In our blinded analysis, all muscles in women who delivered by cesarean section were identified as normal. However, in one case, the puboperinealis muscle was not visible in the 3D EAUS volume but appeared normal in the 3D EVUS. This discrepancy raises the question of whether the absence of this muscle in one imaging modality could be a normal variation. It highlights that these two imaging modalities complement each other, suggesting that they should be used together when evaluating deviations in this region. We have not been able to quantify the degree of the deviations in the muscles in a scoring system. Nevertheless, the excellent reliability of our findings suggests that our protocol can be used as a methodology for further research and clinical practice.

We have previously validated our analysis protocol against pelvic floor symptoms related to a deficient perineum [10, 20, 21]. This highlights that the assessment of the perineal body anatomy using ultrasound has a clinical implication. Using the methodology presented in this paper, we found that women with deviation in at least one of the muscles fusing into the perineal body has a 2.9 higher odds ratio for clinically relevant pelvic floor symptoms such as air trapped inside the vagina, the feeling of a wide vaginal opening, defecation difficulties, and sexual dysfunction after birth [10]. This association remained significant even after adjusting for levator ani avulsions. These symptoms were systematically assessed using KAPTAIN, a validated inventory for evaluating symptoms of a deficient perineal body in women after birth [20]. However, the study lacked statistical power to determine the specific influence of each muscle separately on these symptoms. Consequently, it remains unclear whether each perineal muscle should be investigated separately or if the perineal body should be assessed as an entity [12]. The strong association between ultrasound findings and symptoms published previously, supports our definition of muscle deviation [10]. Regardless, all muscles contributing fibers to the perineal body are vulnerable to severe damage from perineal tears during childbirth, and further research is needed to fully understand sonographic changes in the perineal area after childbirth-related injuries.

### Perineal Body

This is one of the first reliability studies to use both 3D EVUS and 3D EAUS for measuring the perineal body size 1 year postpartum. We found that intra- and inter-rater reliability for perineal body measurements was higher with 3D EAUS than with 3D EVUS. This may be due to the closer and stretched visualization of the perineal body in the 3D EAUS volumes, facilitated by the physiology of the anal canal, which allows for more precise measurements. Additionally, the perineal body measurements were consistently larger in the 3D EAUS volumes compared to the 3D EVUS volumes across both delivery groups. This difference could be attributed to perineal compression caused by probe pressure during the 3D EVUS examination. In contrast, during 3D EAUS acquisition, the probe is stabilized by the anal sphincter and rests on the posterior portion of the puborectalis muscle, minimizing compression effects.

The clinical relevance of perineal body measurements obtained by 3D EVUS and 3D EAUS remains uncertain. However, these imaging modalities may provide valuable complementary information to the POP-Q scoring system, which does not adequately describe the perineal body, as the presence of skin and fat tissue may lead to an overestimation of its size [22, 23]. Previous studies suggest that a smaller perineal body may result from anal sphincter injuries, although less severe perineal tears and vaginal delivery alone can also contribute to its reduction [24, 25].

### Strengths and Limitations

The strengths of this study were that the investigators were blinded to each other’s results and background data. Investigators come from different specialties (colorectal surgery and gynecology), have obtained their ultrasound skills from two independent professional environments, and have different experience levels. However, ideally, an intra-rater reliability analysis should have been performed for the second investigator (MS). Data is presented according to guidelines for reporting reliability and agreement studies [26].

A limitation is the lack of prepartum ultrasound volumes of the participants.

Also, we have interpreted the structures in the perineum from their location according to descriptions in the literature and volumes from study participants who underwent cesarean section, as we did not have any gold standard available from, for example, dissections of cadavers. For this reason, we avoided terms such as “muscle injury” or “damage” and instead described deviations from normal anatomy. Although these deviations are likely related to childbirth trauma, this cannot not be stated with certainty. Ideally the reliability should have been accessed for the entire ultrasound procedure, including volume acquisitions and offline analysis [27]. However, compared to the dynamic volume acquisition during transperineal ultrasound, the 3D EVUS and 3D EAUS acquisitions are static procedures performed at rest which limits the importance of testing the reliability of the acquisition procedure.

As no prior studies have examined deviations in the muscles fusing into the perineal body after birth, a formal sample size calculation was not feasible. Because the kappa statistics are influenced by the prevalence of the outcome and adjusted for chance agreement, high or low prevalence levels can yield misleading kappa values, even when observed agreement is high [28]. To account for this, we strategically selected an equivalent number of participants from both groups: those less likely to show signs of muscle deviation (cesarean section) and those more likely to exhibit such signs (vaginal delivery). Therefore, the number of participants with normal perineal anatomy was higher than a normal childbearing population. Although this is a single-center study, the investigators come from diverse clinical backgrounds and have varying levels of ultrasound experience. Therefore, we expect that our findings can be reproduced by clinicians and researchers in other centers.

## Conclusion

3D EVUS and 3D EAUS are reliable methods for identifying deviations in the transverse perineal, puboperinealis, and puboanalis muscles, as well as for measuring perineal body size 1 year postpartum. Our findings suggest that these two modalities are complementary, with 3D EVUS providing better visualization of the individual muscle insertions, while 3D EAUS offers more reliable measurements of perineal body size.

## Declarations

### Prior Presentations

Parts of this article have been presented at ICS, Toronto, 29September 2023, and NUGA-meeting, Malmø, 2 February 2024
